# Genotype-Specific Biochemical Responses of Apple Trees to Repeated Drought–Rehydration Cycles

**DOI:** 10.3390/plants15172701

**Published:** 2026-09-02

**Authors:** Tomáš Rýgl, František Hnilička, Marek Drímal, Barbora Benická, Lenka Kučírková, Matej Šuránek, Pavol Suran

**Affiliations:** 1 Department of Botany and Plant Physiology, Faculty of Agrobiology, Food and Natural Resources, Czech University of Life Sciences Prague, Kamýcká 129, 165 21 Prague-Suchdol, Czech Republic; ryglt@af.czu.cz; 2 Department of Technology, Faculty of Natural Sciences, Matej Bel University in Banska Bystrica, Tajovského 40, 974 01 Banská Bystrica, Slovakia; marek.drimal@umb.sk; 3 Department of Chemistry, Faculty of Natural Sciences, Matej Bel University in Banska Bystrica, Tajovského 40, 974 01 Banská Bystrica, Slovakia; barbora.benicka@umb.sk (B.B.); matej.suranek@umb.sk (M.Š.); 4 Department of Languages, Faculty of Economics and Management, Czech University of Life Sciences Prague, Kamýcká 129, 165 21 Prague-Suchdol, Czech Republic; kucirkova@pef.czu.cz; 5 Research and Breeding Institute of Pomology Holovousy Ltd., Holovousy 129, 50801 Hořice v Podkrkonoší, Czech Republic; suran@vsuo.cz

**Keywords:** *Malus domestica*, drought stress, drought–rehydration cycles, genotype-specific response, proline, malondialdehyde, antioxidant enzymes, phenolic compounds, principal component analysis

## Abstract

Repeated drought–rehydration episodes expose perennial fruit trees to alternating stress and recovery phases, while treatment-level averages can obscure genotype-specific response strategies. We evaluated seven biochemical markers in fourteen apple (*Malus* *domestica* Borkh.) genotypes during two consecutive drought–rehydration cycles: proline, malondialdehyde (MDA), total phenolics, total flavonoids, and the activities of superoxide dismutase (SOD), catalase (CAT), and peroxidase (POX). Both drought cycles increased all measured markers, with the strongest overall response at the end of the second drought cycle (T6). Relative to the control, mean values at T6 increased by 272.4% for proline, 136.4% for MDA, 45.3% for total phenolics, 76.5% for total flavonoids, 109.1% for SOD, 89.0% for CAT, and 117.5% for POX. Genotype-specific log_2_(Stress/Control) profiles at the first (T2) and second (T6) drought maxima revealed contrasting combinations of osmotic adjustment, oxidative damage, antioxidant-enzyme activity, and secondary-metabolite accumulation. B11 combined comparatively low MDA induction with strong antioxidant-enzyme responses, whereas HL1282 and HL1579 showed lower proline and MDA induction together with stronger phenolic, flavonoid, and selected enzyme responses. In contrast, ‘Idared’ showed strong proline and MDA induction. The first two principal components explained 75.1% of the total variance. Overall, the results identify distinct genotype-specific biochemical response strategies and show that coordinated marker profiles provide substantially more information than individual biomarkers or treatment averages alone.

## 1. Introduction

Climate change is among the most significant environmental challenges affecting contemporary agricultural production. Rising temperatures, changing precipitation patterns, and increasing evapotranspiration are leading to more frequent, prolonged, and severe drought events [1,2,3,4]. Consequently, drought has become one of the major abiotic stresses limiting plant growth, productivity, and yield stability. Its impacts are particularly severe in perennial cropping systems, which are repeatedly exposed to fluctuating environmental conditions over multiple growing seasons [5,6,7]. Unlike annual crops, which may escape drought by completing their life cycle [8], long-lived fruit trees must rely on persistent physiological and biochemical mechanisms, including root system development, stomatal regulation, osmotic adjustment, and antioxidant protection, to withstand water deficit and subsequently recover [8].

Apple (*Malus domestica* Borkh.) is one of the most economically important fruit crops in temperate regions. According to FAOSTAT (2026) [9], apple orchards occupy approximately 4.68 million ha worldwide, with an average yield of 20.89 t ha^−1^. In Europe, apple cultivation covers approximately 0.88 million ha, with an average yield of 19.16 t ha^−1^. These figures refer to 2024 data. A substantial share of commercial apple production is located in temperate and Mediterranean regions where increasingly irregular precipitation, high evaporative demand, and seasonal irrigation requirements expose orchards to recurrent water limitation [10,11,12]. Recent orchard studies reinforce the practical relevance of this constraint: severe irrigation deprivation in a Mediterranean high-density apple orchard caused profound physiological impairment and ultimately tree mortality, while research in Central European commercial orchards identifies declining water availability and more frequent dry summer periods as increasing challenges for stable apple production [13,14]. Vegetative growth, fruit development, and long-term orchard productivity are therefore increasingly affected by reduced or less predictable water availability [9,10,11,12,13,14]. Considerable variability exists among apple genotypes and rootstocks in root system architecture, hydraulic conductivity, stomatal regulation, osmotic adjustment, and antioxidant capacity, and the widely used M9 rootstock is considered particularly sensitive to drought stress [15,16,17]. Identifying traits associated with enhanced drought tolerance is therefore important for breeding programmes and for selecting plant material suitable for water-limited environments.

Water deficit rapidly decreases leaf water potential and turgor, leading to partial or complete stomatal closure. Restricted CO_2_ uptake subsequently reduces photosynthetic carbon assimilation and disrupts the balance between absorbed and metabolically utilised light energy [18,19]. Consequently, drought promotes excessive production of reactive oxygen species (ROS) and disturbs cellular redox homeostasis. Under physiological conditions, ROS function as important signalling molecules; however, their excessive accumulation damages membranes, proteins, nucleic acids, and photosynthetic pigments [20,21,22]. As reviewed by Samanta et al. [23], ROS and reactive nitrogen species (RNS) interact with phytohormone signalling pathways and form an integrated regulatory network involved in plant responses to drought stress. Drought tolerance therefore depends not on the complete elimination of ROS but on the ability to regulate their production and detoxification while maintaining their signalling functions and preventing excessive oxidative damage [20,21,22,23,24].

The enzymatic antioxidant defence system comprises superoxide dismutase (SOD), catalase (CAT), and various peroxidases (POX). SOD catalyses the dismutation of superoxide radicals into hydrogen peroxide, which is subsequently detoxified mainly by catalase and peroxidases [25,26,27]. However, the efficiency of antioxidant protection depends not on the activity of a single enzyme but on the coordinated action of multiple antioxidant enzymes operating in different cellular compartments and at different stages of stress response [24,25,26,27]. Elevated SOD activity without a corresponding capacity to remove H_2_O_2_ may even aggravate oxidative imbalance. Antioxidant enzymes should therefore be considered components of an integrated functional defence network.

Enzymatic antioxidant defence is complemented by non-enzymatic mechanisms involved in osmotic adjustment, membrane stabilisation, and redox signalling. Proline is one of the most extensively studied compatible osmolytes. Its accumulation lowers osmotic potential, promotes water retention, stabilises proteins and membranes, and contributes to ROS regulation and metabolic recovery following rehydration [8,28,29,30,31]. In contrast, malondialdehyde (MDA), produced during membrane lipid peroxidation, is widely used as an indicator of oxidative damage and loss of membrane integrity [24]. The combined assessment of proline and MDA therefore provides insight into the balance between protective adaptation and stress-induced cellular damage.

Phenolic compounds and flavonoids represent another important component of non-enzymatic defence. These secondary metabolites can scavenge ROS, chelate transition metals, stabilise cellular membranes, and modulate signalling pathways involved in stress acclimation [32,33]. Their accumulation is generally associated with activation of the phenylpropanoid pathway, although both the magnitude and temporal dynamics of this response vary considerably among genotypes [34,35]. Consequently, phenolic compounds and flavonoids provide complementary information on metabolic plasticity and the capacity of plants to cope with repeated drought stress [36,37,38,39].

Under natural conditions, drought rarely occurs as a single isolated event. Instead, periods of water deficit are interrupted by rainfall or irrigation, resulting in repeated cycles of dehydration and rehydration during the growing season. Rehydration is not merely a passive return to the initial physiological state but an active process involving repair of the photosynthetic apparatus, restoration of redox homeostasis, redistribution of osmolytes, and reorganisation of antioxidant metabolism [28,29,30,31,39]. Previous exposure to drought may also induce stress memory, enabling faster or more efficient activation of defence mechanisms during subsequent drought events [40,41]. Conversely, incomplete recovery between stress episodes may result in cumulative physiological and biochemical damage.

Although numerous studies have investigated drought responses in apple trees [42,43,44,45,46], most have focused on physiological traits or on a limited number of biochemical markers and have generally evaluated a single drought episode. Consequently, genotype-specific coordination of osmotic adjustment, oxidative damage, enzymatic antioxidant defence, and secondary metabolism during repeated drought–rehydration cycles remains insufficiently resolved. Broad treatment averages may further mask contrasting response strategies among genotypes. Multivariate approaches, particularly principal component analysis (PCA), correlation analysis, and hierarchical clustering, can integrate complementary biochemical traits and help reveal such genotype-dependent patterns [47,48].

The aim of this study was therefore to characterise genotype-specific biochemical responses of fourteen apple genotypes during two consecutive drought–rehydration cycles using a defined panel of seven complementary markers. In addition to describing overall temporal dynamics, we specifically compared genotype-level responses at the first and second drought maxima and evaluated whether repeated stress altered the relative contribution of osmotic adjustment, oxidative damage, antioxidant-enzyme activity, and secondary metabolism. We hypothesised that repeated drought would generate distinct biochemical response profiles among genotypes rather than a uniform scaling of all measured markers.

## 2. Results

### 2.1. Overall Temporal Dynamics of the Biochemical Response

**Figure 1:**
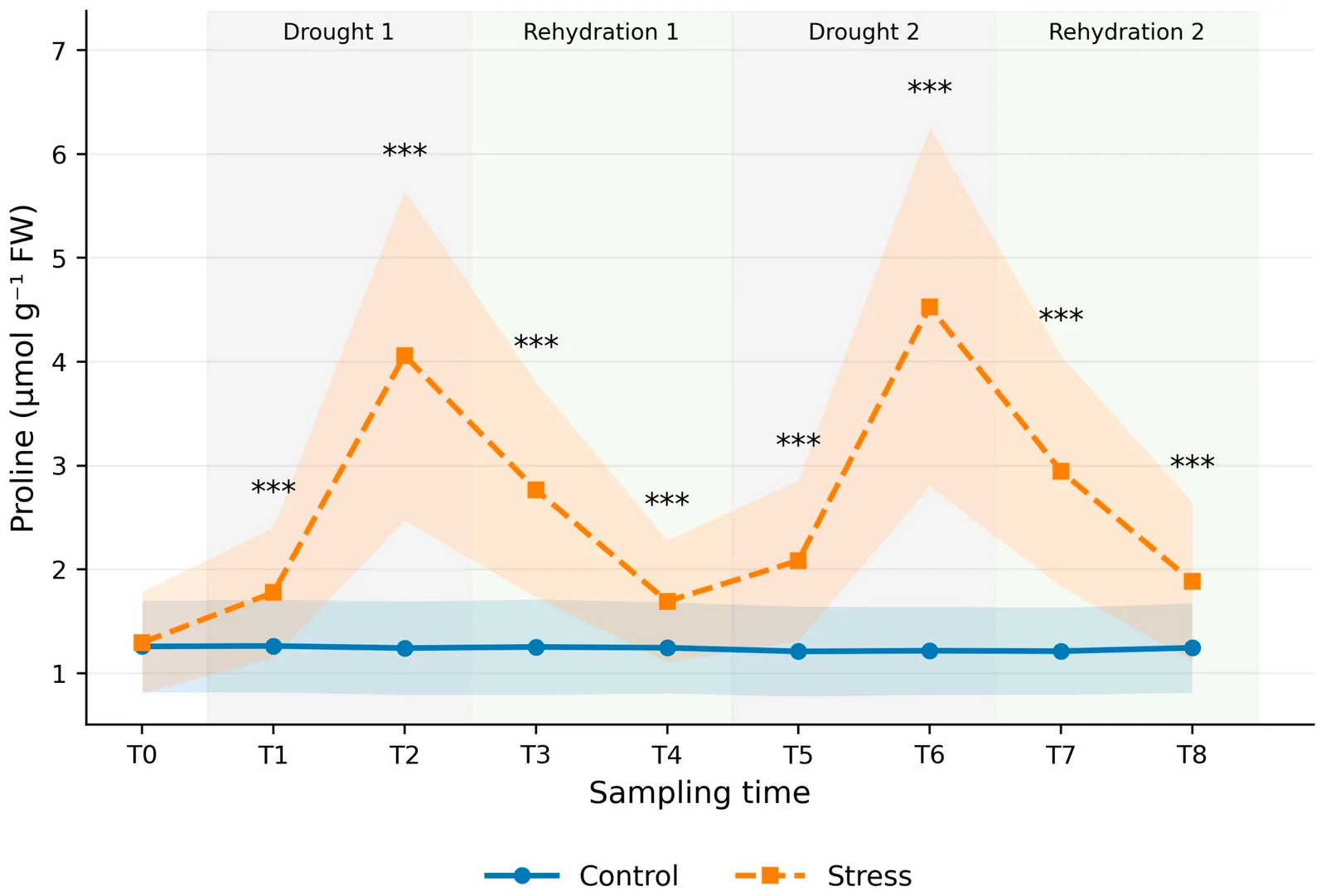
Temporal dynamics of proline content in control and drought-stressed apple plants during two drought–rehydration cycles. Values represent means ± SD of 14 genotype means; each genotype mean was calculated from five biological replicates. Asterisks indicate significant differences between control and stress treatments at individual sampling times (Tukey’s HSD: *** *p* < 0.001). The shaded background areas distinguish the experimental phases: the drought periods (Drought 1 and Drought 2) and the rehydration periods (Rehydration 1 and Rehydration 2).

**Figure 2:**
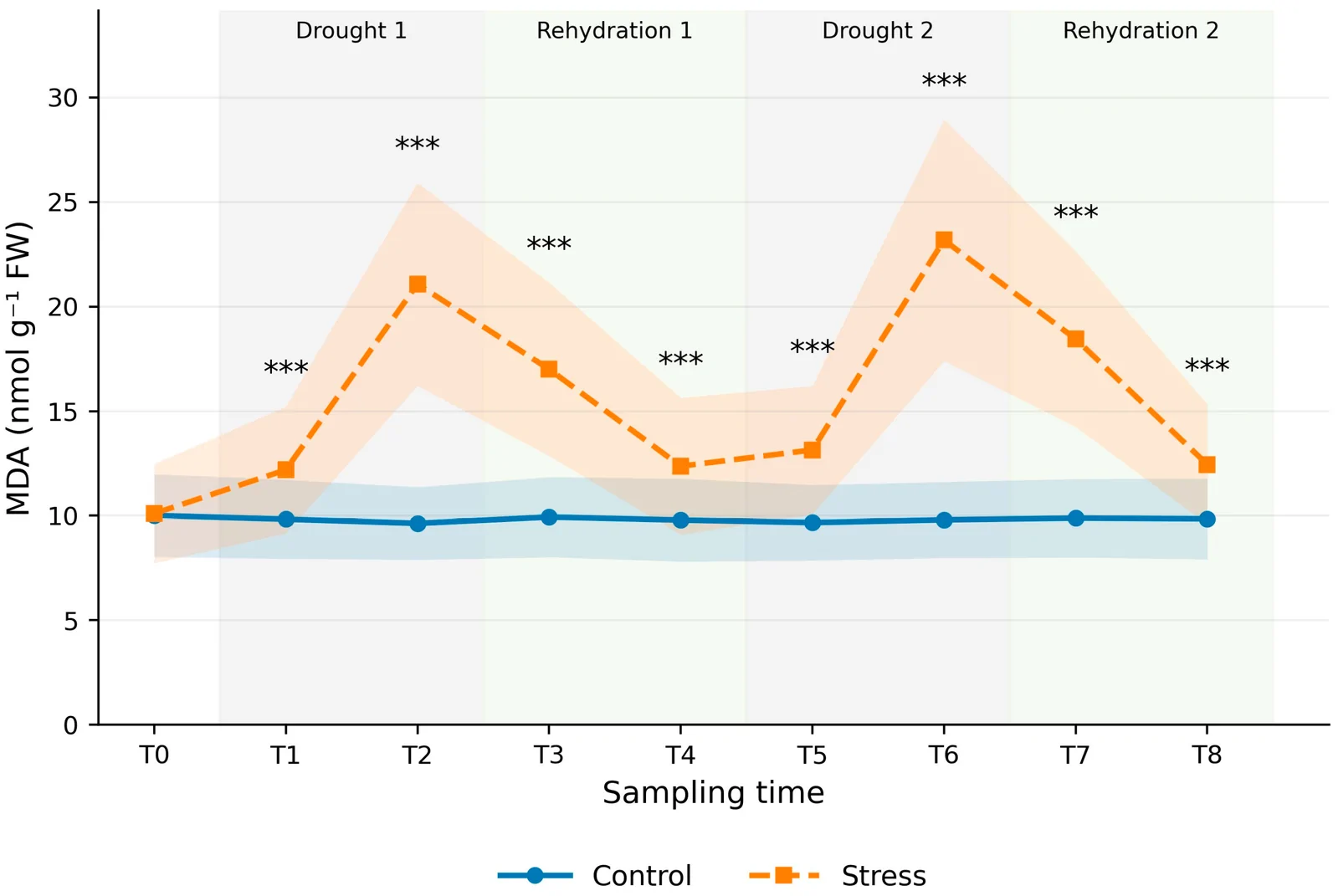
Temporal dynamics of malondialdehyde (MDA) content in control and drought-stressed apple plants during two drought–rehydration cycles. Values and significance notation are described in Figure 1 (*** *p* < 0.001). The shaded background areas distinguish the experimental phases: the drought periods (Drought 1 and Drought 2) and the rehydration periods (Rehydration 1 and Rehydration 2).

**Figure 3:**
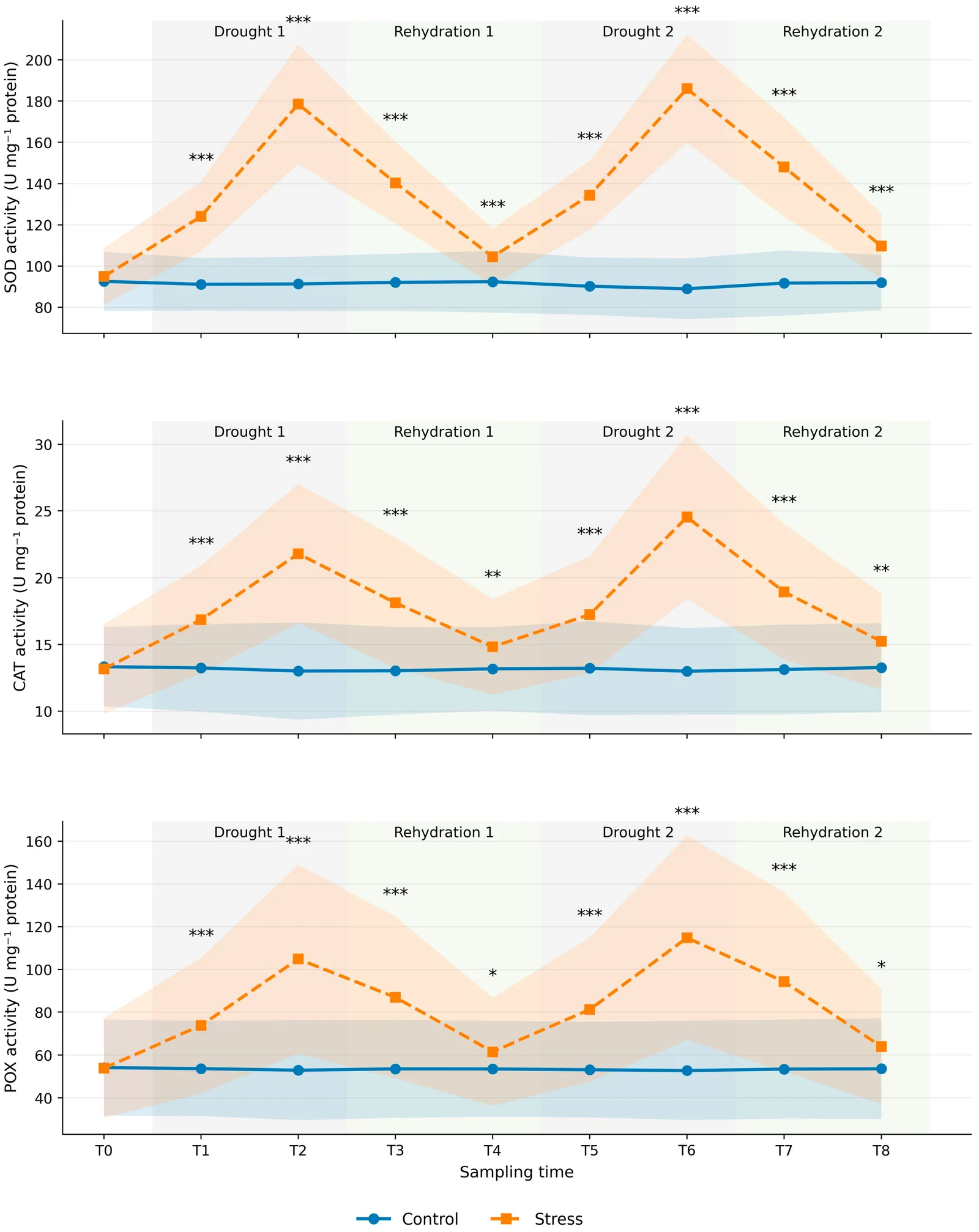
Temporal dynamics of antioxidant enzyme activities: superoxide dismutase (SOD), catalase (CAT), and peroxidase (POX). Values represent means ± SD of 14 genotype means; each genotype mean was calculated from five biological replicates. Asterisks denote significant differences between control and stress treatments at individual sampling times (Tukey’s HSD, * *p* < 0.05, ** *p* < 0.01, *** *p* < 0.001). The shaded background areas distinguish the experimental phases: the drought periods (Drought 1 and Drought 2) and the rehydration periods (Rehydration 1 and Rehydration 2).

**Figure 4:**
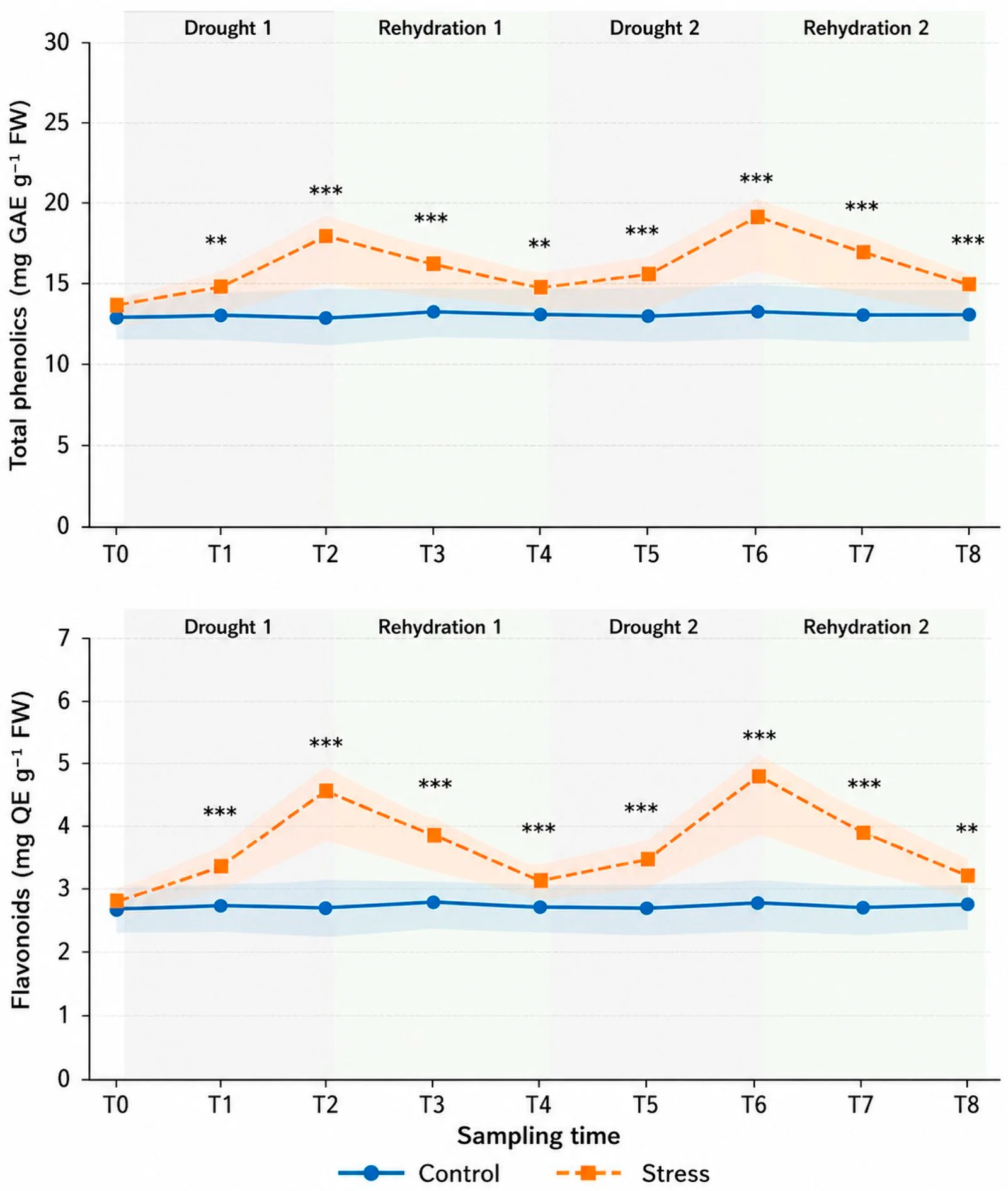
Temporal dynamics of total phenolics and flavonoids in control and drought-stressed apple plants. Values represent means ± SD of 14 genotype means; each genotype mean was calculated from five biological replicates. Asterisks denote significant differences between treatments (Tukey’s HSD, ** *p* < 0.01, *** *p* < 0.001). The shaded background areas distinguish the experimental phases: the drought periods (Drought 1 and Drought 2) and the rehydration periods (Rehydration 1 and Rehydration 2).

At the baseline sampling point (T0), the values of all seven biochemical markers were comparable between the control and drought-stressed treatments, with no significant differences detected. In the control treatment, the biochemical parameters remained relatively stable throughout the experiment, whereas water deficit induced a marked time-dependent response in the drought-stressed plants (Figure 1, Figure 2, Figure 3 and Figure 4).

The first pronounced increase in biochemical activity was observed during the first drought cycle, with most parameters reaching their first maximum at T2. Subsequent rehydration (T3–T4) resulted in a decline in all measured parameters, although their values generally remained above those of the corresponding controls. Re-exposure to water deficit induced a second increase, and the highest mean values of all seven markers were recorded at T6, at the end of the second drought cycle (Figure 1, Figure 2, Figure 3 and Figure 4). During the second rehydration period (T7–T8), the values decreased again, but complete recovery to control levels was not observed for most parameters. Genotype-specific differences underlying these treatment-level trends are analysed explicitly in Section 2.7. 

At T6, proline content increased from 1.216 ± 0.435 to 4.528 ± 1.726 µmol g^−1^ FW, MDA from 9.804 ± 2.068 to 23.175 ± 5.939 nmol g^−1^ FW, total phenolics from 13.541 ± 2.809 to 19.673 ± 4.057 mg GAE g^−1^ FW, and flavonoids from 2.807 ± 0.674 to 4.955 ± 1.075 mg QE g^−1^ FW in the control and drought-stressed treatments, respectively. SOD activity increased from 88.934 ± 15.938 to 186.001 ± 30.217 U mg^−1^ protein, CAT from 12.988 ± 3.428 to 24.548 ± 6.478 U mg^−1^ protein, and POX from 52.772 ± 22.855 to 114.790 ± 47.600 U mg^−1^ protein.

Three-way ANOVA showed significant effects of genotype, treatment, and sampling date on all biochemical parameters, together with significant two- and three-way interactions (Table S1).

### 2.2. Osmotic Adjustment and Membrane Lipid Peroxidation

Proline showed one of the strongest responses to water deficit (Figure 1). At T1, its mean content increased from 1.262 ± 0.449 µmol g^−1^ FW in the control to 1.776 ± 0.633 µmol g^−1^ FW under water deficit, corresponding to an increase of 40.7%. At the end of the first drought cycle (T2), proline reached 4.059 ± 1.591 µmol g^−1^ FW, compared with 1.242 ± 0.453 µmol g^−1^ FW in the control, representing an increase of 227.0%.

During the first rehydration period, proline content declined and reached 1.691 ± 0.597 µmol g^−1^ FW at T4, compared with 1.244 ± 0.446 µmol g^−1^ FW in the control. Following the onset of the second drought cycle, proline accumulated again and reached its maximum at T6 (4.528 ± 1.726 µmol g^−1^ FW), representing a 272.4% increase relative to the control (1.216 ± 0.435 µmol g^−1^ FW). After the second rehydration, proline declined to 1.882 ± 0.764 µmol g^−1^ FW at T8, whereas the corresponding control value was 1.244 ± 0.430 µmol g^−1^ FW.

MDA exhibited a similar temporal pattern (Figure 2). At T1, MDA increased from 9.832 ± 2.027 to 12.192 ± 3.134 nmol g^−1^ FW, and at T2 it reached 21.073 ± 5.015 nmol g^−1^ FW, compared with 9.632 ± 1.840 nmol g^−1^ FW in the control. Following the first rehydration, MDA declined to 12.368 ± 3.436 nmol g^−1^ FW at T4.

The highest MDA content was observed at T6, when the drought-stressed treatment reached 23.175 ± 5.939 nmol g^−1^ FW, compared with 9.804 ± 2.068 nmol g^−1^ FW in the control, corresponding to an increase of 136.4%. At T8, MDA decreased to 12.451 ± 2.983 nmol g^−1^ FW, whereas the control value was 9.851 ± 2.036 nmol g^−1^ FW.

Three-way ANOVA revealed strong effects of genotype, treatment, and sampling date on both parameters (Table S1). For proline, partial η^2^ values were 0.916, 0.914, and 0.870 for genotype, treatment, and sampling date, respectively. For MDA, the corresponding values were 0.821, 0.855, and 0.764. Significant two- and three-way interactions were also detected. Genotype-specific temporal responses are provided in Figures S1 and S2, with detailed values in Table S2.

### 2.3. Enzymatic Antioxidant Defence

The activities of SOD, CAT, and POX increased during both drought cycles and declined following rehydration (Figure 3). At T2, SOD activity reached 178.541 ± 31.812 U mg^−1^ protein in drought-stressed plants compared with 91.295 ± 14.716 U mg^−1^ protein in the control. CAT activity reached 21.802 ± 5.318 compared with 13.003 ± 3.759 U mg^−1^ protein, whereas POX activity reached 104.855 ± 44.062 compared with 52.936 ± 23.210 U mg^−1^ protein.

The highest activities of all three enzymes were recorded at T6. SOD activity reached 186.001 ± 30.217 U mg^−1^ protein, compared with 88.934 ± 15.938 U mg^−1^ protein in the control, representing an increase of 109.1%. CAT activity increased by 89.0%, from 12.988 ± 3.428 to 24.548 ± 6.478 U mg^−1^ protein, while POX showed the largest relative increase among the three enzymes, rising by 117.5%, from 52.772 ± 22.855 to 114.790 ± 47.600 U mg^−1^ protein.

Following the second rehydration, enzyme activities declined. At T8, SOD, CAT, and POX activities in the drought-stressed treatment were 109.671 ± 17.750, 15.221 ± 3.858, and 63.889 ± 26.870 U mg^−1^ protein, respectively. Three-way ANOVA showed significant effects of genotype, treatment, and sampling date on all three enzyme activities (*p* < 0.001; Table S1). For SOD, partial η^2^ values were 0.663 for genotype, 0.810 for treatment, and 0.647 for sampling date. For CAT, the corresponding values were 0.860, 0.720, and 0.551, respectively. POX exhibited the strongest genotype effect among the enzymatic markers (partial η^2^ = 0.944), while the effects of treatment and sampling date were 0.817 and 0.670, respectively. Significant two- and three-way interactions were also observed. Genotype-specific temporal responses of SOD, CAT, and POX are shown in Figures S3–S5.

### 2.4. Total Phenolics and Flavonoids

Water deficit also increased the contents of total phenolics and flavonoids (Figure 4). At T2, total phenolic content reached 18.504 ± 4.039 mg GAE g^−1^ FW in the drought-stressed treatment compared with 13.247 ± 2.803 mg GAE g^−1^ FW in the control. Flavonoid content increased to 4.555 ± 0.970 mg QE g^−1^ FW, compared with 2.746 ± 0.657 mg QE g^−1^ FW in the control.

Both parameters declined during the first rehydration period. At T4, total phenolics and flavonoids in drought-stressed plants reached 14.914 ± 3.170 mg GAE g^−1^ FW and 3.117 ± 0.700 mg QE g^−1^ FW, respectively.

The highest values were recorded at T6. Total phenolic content reached 19.673 ± 4.057 mg GAE g^−1^ FW, compared with 13.541 ± 2.809 mg GAE g^−1^ FW in the control, representing an increase of 45.3%. Flavonoid content increased by 76.5%, from 2.807 ± 0.674 to 4.955 ± 1.075 mg QE g^−1^ FW. Following the second rehydration, the values declined to 15.126 ± 2.993 mg GAE g^−1^ FW for total phenolics and 3.160 ± 0.733 mg QE g^−1^ FW for flavonoids at T8.

The genotype effect was strong for both total phenolics (partial η^2^ = 0.857) and flavonoids (0.889). The treatment effect reached 0.612 for total phenolics and 0.797 for flavonoids, whereas the sampling-date effect was 0.394 and 0.672, respectively (Table S1). Significant interactions among the experimental factors were also detected. Genotype-specific temporal responses of total phenolics and flavonoids are shown in Figures S6 and S7.

### 2.5. Relationships Among Biochemical Markers

**Figure 5:**
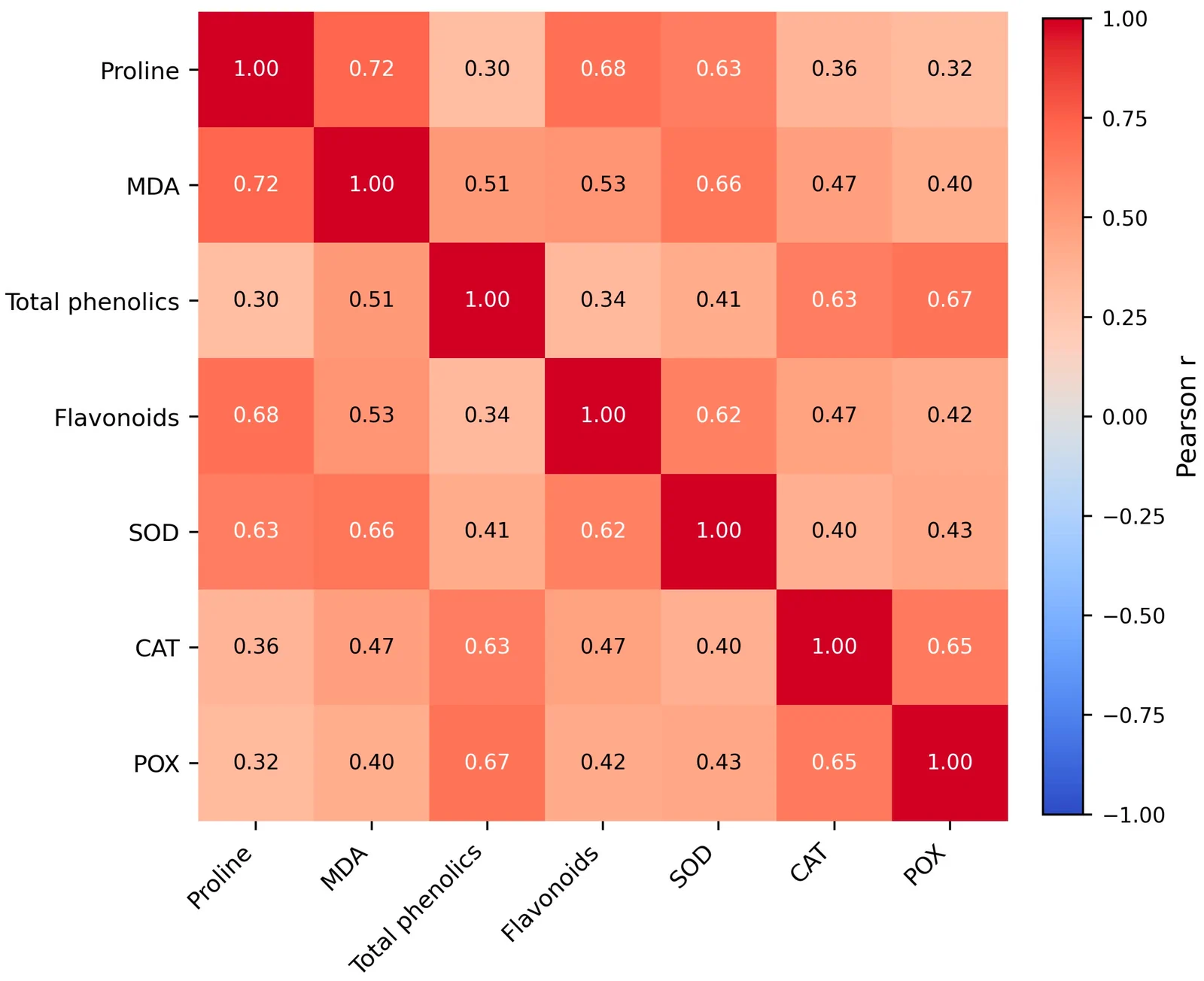
Pearson correlation matrix among seven biochemical markers across the experimental dataset. Values in cells are Pearson correlation coefficients (r).

Pearson’s correlation analysis revealed predominantly positive relationships among the biochemical markers (Figure 5). The strongest correlation was observed between proline and MDA (r = 0.724). Proline was also positively correlated with flavonoids (r = 0.684) and SOD (r = 0.629).

MDA was positively correlated with SOD (r = 0.663), flavonoids (r = 0.527), and total phenolics (r = 0.507). Total phenolics showed the strongest correlations with POX (r = 0.672) and CAT (r = 0.632). Positive relationships were also observed between CAT and POX (r = 0.646) and between flavonoids and SOD (r = 0.621).

Overall, the correlation matrix showed that changes in osmotic adjustment, lipid peroxidation, secondary metabolism, and antioxidant enzyme activities occurred in a coordinated manner during the experimental period (Figure 5). Exploratory genotype-wise correlations between proline and MDA were also positive for all fourteen genotypes (r = 0.887–0.956), including B11 (r = 0.904), indicating that the positive association was not restricted to a subset of genotypes.

### 2.6. Principal Component Analysis

**Figure 6:**
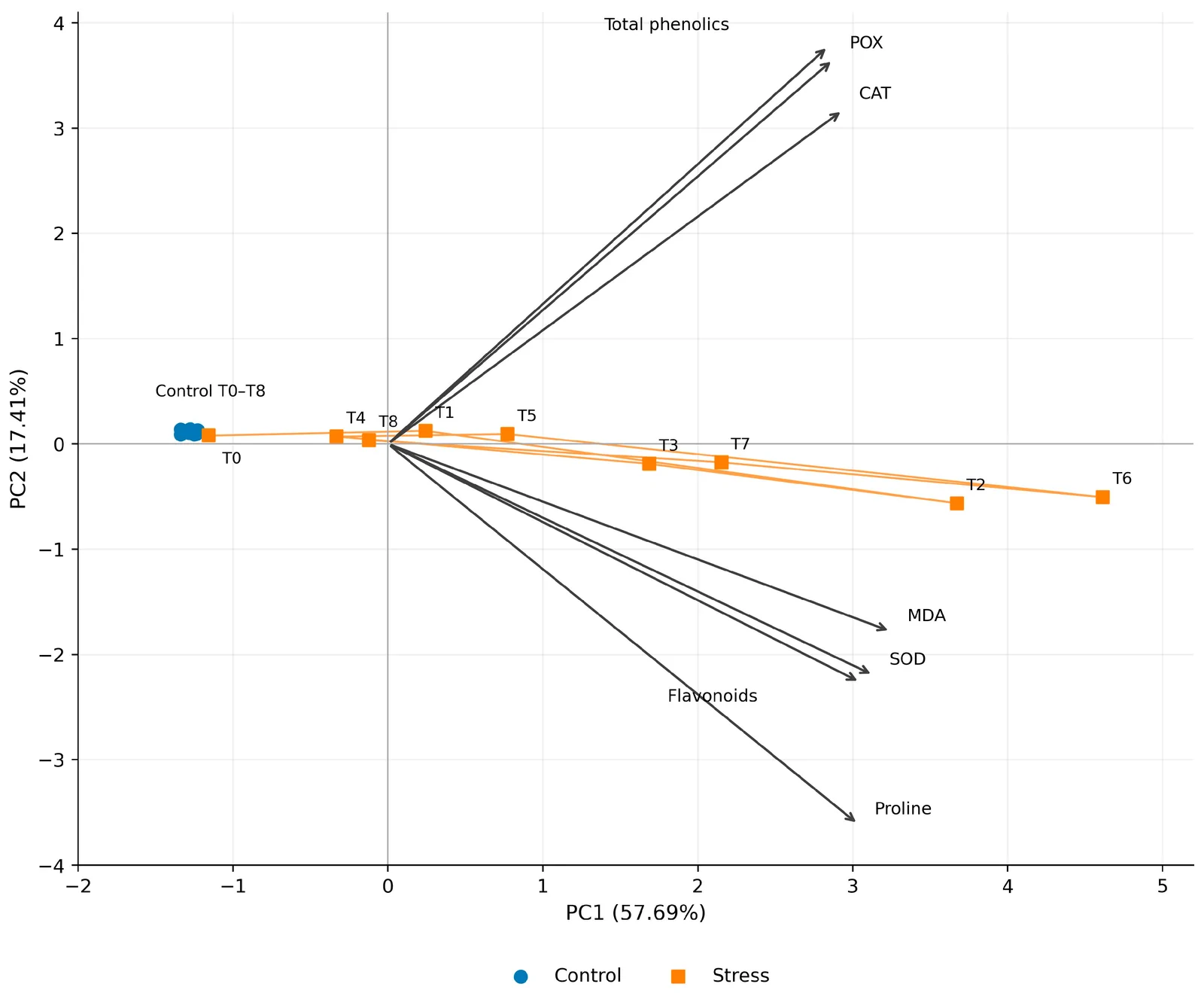
Principal component analysis (PCA) biplot of biochemical responses during repeated drought and rehydration. PC1 and PC2 explain 57.69% and 17.41% of the total variance, respectively. Points represent treatment × sampling-time centroids; arrows indicate variable loadings.

Principal component analysis was performed using standardized values of the seven biochemical parameters. The first two principal components explained 75.10% of the total variance, with PC1 accounting for 57.69% and PC2 for 17.41% (Figure 6).

All biochemical markers had positive loadings on PC1. The highest loadings were observed for MDA (0.406), SOD (0.392), flavonoids (0.381), and proline (0.380), followed by CAT (0.368), POX (0.360), and total phenolics (0.356). Thus, PC1 represented a common gradient associated with the overall magnitude of the biochemical response.

PC2 was characterized by positive loadings of total phenolics (0.473), POX (0.457), and CAT (0.397) and a pronounced negative loading of proline (−0.452). Flavonoids (−0.283), SOD (−0.275), and MDA (−0.223) also showed negative loadings on PC2. Numerical PCA loadings and explained variance are provided in Table S3.

The distribution of samples in the PCA space reflected the temporal progression of the experiment. Samples collected during the drought maxima, particularly T2 and T6, were separated from the control and rehydration stages along PC1, whereas rehydrated samples generally occupied intermediate positions (Figure 6).

### 2.7. Genotype-Specific Responses at the First and Second Drought Maxima

**Figure 7:**
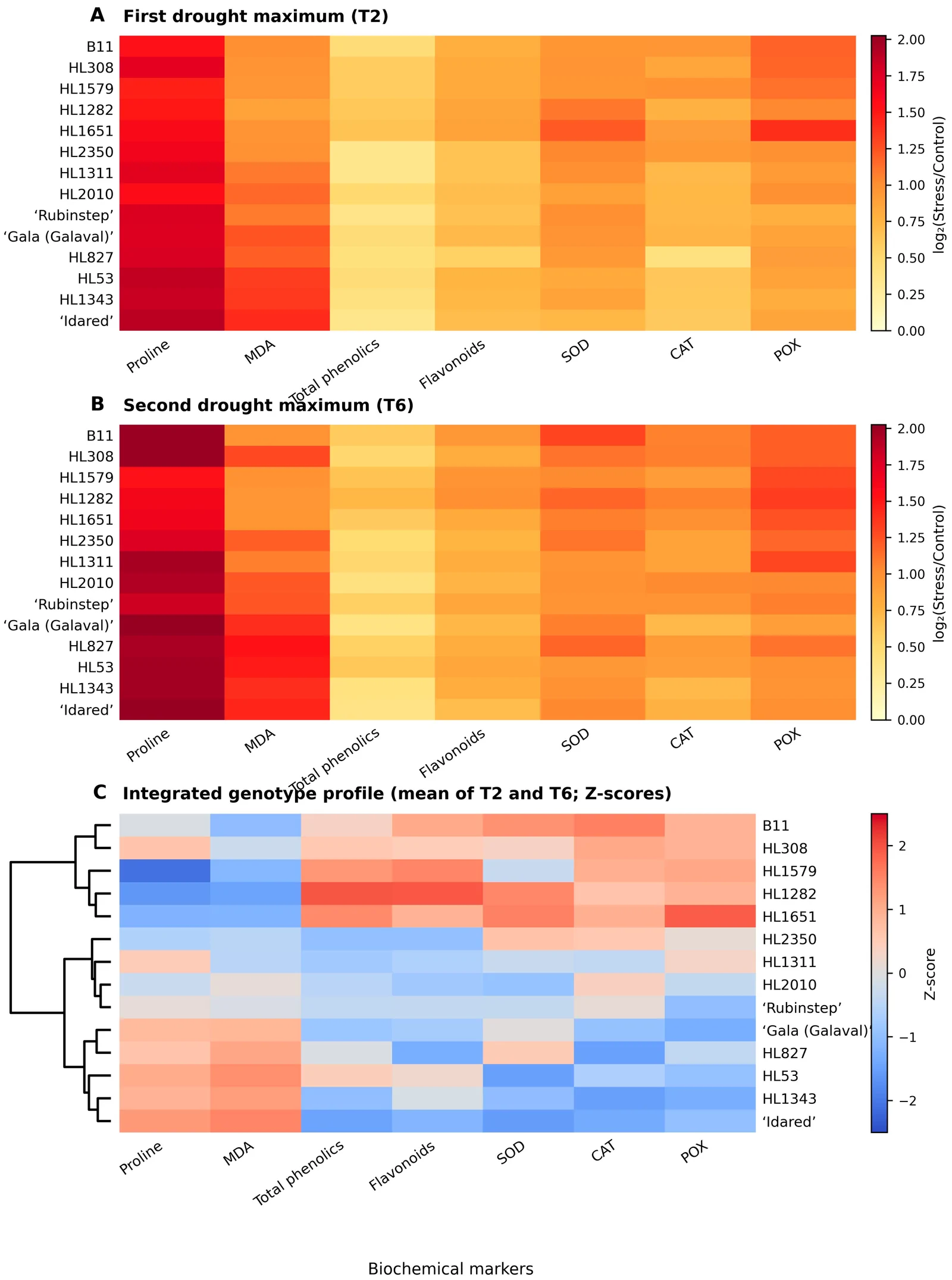
Genotype-specific biochemical response profiles at the two drought maxima. Panels (**A**,**B**) show log_2_(Stress/Control) responses at T2 and T6, respectively. Panel (**C**) shows the mean of the T2 and T6 responses, standardised as Z-scores and clustered using Euclidean distance and Ward’s method. Warmer colours in (**A**,**B**) indicate stronger stress-induced increases.

To resolve differences that were obscured by treatment-level averages, genotype-specific responses were examined separately at the end of the first (T2) and second (T6) drought periods. For each genotype and marker, the response was expressed as log_2_(Stress/Control), allowing direct comparison of the magnitude and composition of biochemical induction across genotypes and between drought cycles (Figure 7A,B). Integrated profiles based on the mean T2 and T6 responses were subsequently standardised as Z-scores and subjected to hierarchical clustering using Euclidean distance and Ward’s method (Figure 7C).

At T2, all seven markers were induced by water deficit in all genotypes, but the magnitude and composition of the response differed substantially. Proline induction was particularly strong in ‘Idared’, HL53, HL1343, and HL827, whereas B11, HL1282, and HL1579 showed comparatively lower proline responses. MDA induction was greatest in ‘Idared’, HL1343, and HL53, while HL1282, HL1579, HL1651, B11, and HL2350 showed lower relative MDA responses. Antioxidant and secondary-metabolite responses did not follow the same ranking, demonstrating that genotypic differences could not be described by a single marker.

At T6, the second drought maximum generally intensified the biochemical response, but the degree of amplification was genotype-dependent. B11 showed a marked further increase in proline and SOD while maintaining comparatively low MDA induction. HL827 showed one of the strongest increases in MDA between T2 and T6 together with increased CAT and POX responses. HL1282 maintained relatively low proline and MDA induction while showing strong phenolic, flavonoid, SOD, CAT, and POX responses. In contrast, ‘Idared’ remained characterised by strong proline and MDA induction relative to several antioxidant-related markers. These differences indicate that repeated drought altered the balance among biochemical response components rather than simply scaling all markers proportionally.

Hierarchical clustering of the integrated T2–T6 profiles separated the fourteen genotypes into four major groups. Cluster 1 comprised B11 and HL308 and was characterised by comparatively strong antioxidant responses relative to MDA induction. Cluster 2 included HL1282, HL1579, and HL1651 and was distinguished by lower relative proline and MDA induction together with stronger phenolic, flavonoid, and antioxidant-enzyme responses. Cluster 3 consisted of HL1311, HL2010, HL2350, and ‘Rubinstep’ and showed predominantly intermediate profiles.

Cluster 4 comprised ‘Gala (Galaval)’, HL1343, HL53, HL827, and ‘Idared’. These genotypes generally showed greater relative induction of proline and MDA combined with weaker responses of several antioxidant-related parameters. The contrast between clusters was therefore driven not by the absolute magnitude of a single response but by different combinations of osmotic adjustment, oxidative damage, enzymatic defence, and secondary metabolism.

Overall, the genotype-specific analysis revealed substantial variation in both the magnitude and composition of the biochemical response to repeated water deficit. These profiles describe distinct biochemical response strategies and should not by themselves be interpreted as direct measures of drought tolerance. Bootstrap consensus analysis provided additional support for the four-cluster solution: mean within-cluster co-clustering probabilities were 0.70 for Cluster 1, 0.83 for Cluster 2, 0.74 for Cluster 3, and 0.70 for Cluster 4 across 1000 resamples. Thus, the strongest support was obtained for Cluster 2, while the remaining groups showed moderate-to-good stability. Detailed genotype-specific temporal trajectories are provided in Figures S1–S7; standardised integrated profiles and cluster membership are provided in Table S4, and bootstrap cluster-stability results in Table S5.

## 3. Discussion

### 3.1. Repeated Water Deficit Intensifies the Biochemical Response

The present study demonstrates that repeated water deficit elicits both a common temporal response and substantial genotype-dependent variation in the relative contribution of individual biochemical processes. Across the full set of fourteen genotypes, drought increased proline, MDA, total phenolics, total flavonoids, SOD, CAT, and POX, and the highest treatment means were recorded at T6. However, the genotype-specific analysis shows that this common temporal trend does not imply a uniform biochemical strategy: the relative balance among osmotic adjustment, oxidative damage, antioxidant enzymes, and secondary-metabolite accumulation differed markedly among genotypes and, for several genotypes, between the first and second drought maxima.

The stronger overall response during the second cycle may reflect the altered physiological and metabolic state created by the first drought–rehydration episode [40,41,49]. Repeated stress can induce stress memory or priming, but recent critical appraisal has emphasised that such interpretations require careful control for developmental and experimental confounders [50]. In the present experiment, most markers remained above control values even after the 14-day first rehydration period under restored full irrigation. Accordingly, the stronger T6 response cannot be attributed unambiguously to beneficial priming and is more appropriately interpreted as the combined outcome of renewed defence activation and residual effects of the preceding drought cycle.

This distinction is essential when interpreting the measured markers. Increased antioxidant-enzyme activity or accumulation of proline and secondary metabolites may contribute to protection, but strong induction can also indicate greater stress exposure [8,25,26,27,51]. Conversely, MDA reflects membrane lipid peroxidation and therefore provides information about oxidative damage occurring alongside defence activation [24,25,26,27,52]. The strength of the present marker-based approach lies in integrating these complementary responses across time and genotypes. Recent apple studies likewise support the view that drought performance emerges from coordinated, genotype-dependent physiological and biochemical processes rather than from a single marker [42,43,44,45,46]. The resulting profiles are therefore interpreted as coordinated biochemical response strategies rather than as a complete molecular description of drought tolerance.

### 3.2. Proline as a Marker of Osmotic Adjustment and Stress Intensity

Among the measured markers, proline exhibited the largest relative treatment response. Its accumulation increased during both drought cycles, reached a maximum at T6, and declined after irrigation was restored. This behaviour is consistent with the established role of proline as a compatible osmolyte involved in osmotic adjustment and in the stabilisation of proteins and cellular membranes under dehydration [8,53].

The decline in proline during rehydration is also biologically informative because accumulated proline can be remobilised as a source of carbon, nitrogen, and reducing equivalents during metabolic recovery [8,53,54,55]. Nevertheless, proline did not return fully to control levels after rehydration, indicating incomplete biochemical recovery or persistence of an elevated osmolyte pool before the subsequent drought episode. The genotype-specific profiles further show that the magnitude of proline induction was not uniform across the material examined.

Importantly, proline was positively correlated with MDA (r = 0.724), showing that greater proline accumulation was not associated with lower oxidative damage across the experimental dataset. This observation is particularly relevant to genotype evaluation: ‘Idared’, for example, showed strong induction of both proline and MDA at the drought maxima, whereas B11 combined strong proline induction with comparatively lower MDA induction. Proline should therefore not be treated as an independent proxy for drought tolerance; its interpretation depends on the accompanying level of oxidative damage, antioxidant activation, and subsequent recovery [8,53,54,55,56,57]. Proline metabolism is also dynamically coupled to mitochondrial redox metabolism. During stress relief, oxidation through proline dehydrogenase can provide reducing equivalents for respiration, whereas excessive or imbalanced Pro–P5C cycling may promote mitochondrial ROS formation. Exploratory genotype-wise correlations were positive in all fourteen genotypes (r = 0.887–0.956), including B11 (r = 0.904), indicating that the overall proline–MDA association does not break down in this genotype. However, the present data do not support a discrete proline threshold separating protective from damage-associated accumulation. Thus, high proline may reflect both protective osmotic adjustment and the intensity of metabolic perturbation rather than protection alone [56,57].

### 3.3. Coordination of Enzymatic Antioxidant Defence

The parallel increases in SOD, CAT, and POX indicate coordinated activation of enzymatic antioxidant defence. SOD converts superoxide radicals to H_2_O_2_, whereas catalase and peroxidases contribute to H_2_O_2_ removal; consequently, the balance among these activities is more informative than the response of any single enzyme [25,26,27]. As discussed by Samanta et al. [23], this coordination operates within a broader redox-signalling network in which ROS and reactive nitrogen species (RNS) are produced in multiple cellular compartments and participate in stress signalling as well as oxidative damage; the biological outcome therefore depends on the spatial and temporal balance between reactive-species production and scavenging. The genotype-specific heat maps support this interpretation by showing that individual genotypes differed not only in the magnitude of enzyme induction but also in the relative contribution of SOD, CAT, and POX between T2 and T6.

Genotype, treatment, and sampling date significantly affected all three enzyme activities. The genotype effect was especially strong for POX (partial η^2^ = 0.944), indicating that variation in this component of antioxidant metabolism was strongly associated with genetic background. The particularly high genotype specificity of POX is biologically plausible because plant class III peroxidases form a large multigene family with broad substrate specificity and functions extending beyond H_2_O_2_ removal to phenolic oxidation, lignification, cell-wall remodelling, and stress signalling [58,59]. Genotype-dependent regulation of different POX isoforms and substrates may therefore generate greater between-genotype variability than observed for the more functionally constrained SOD and CAT assays. At the drought maxima, genotypes such as B11, HL1282, HL1579, and HL1651 showed relatively strong induction of one or more antioxidant enzymes, whereas other genotypes displayed profiles dominated more strongly by proline and/or MDA.

POX may also be functionally linked to phenolic metabolism, lignification, and cell-wall modification [25,26,27,35,59]. The positive correlation between total phenolics and POX is therefore biologically plausible and suggests coordination between enzymatic antioxidant processes and phenolic-associated metabolism. However, the present data do not resolve specific phenolic substrates or individual peroxidase isoforms, and the correlation should consequently be interpreted as an association between broad response components rather than evidence for a defined biochemical pathway.

APX activity was not determined, and the study therefore does not characterise the complete ascorbate–glutathione cycle. The SOD, CAT, and POX measurements instead provide a focused comparison of major antioxidant-enzyme responses across genotypes and across repeated drought and rehydration.

### 3.4. Phenolic Compounds and Flavonoids as Components of the Longer-Term Antioxidant Response

Total phenolic and total flavonoid contents increased during water deficit and generally declined more slowly than proline during rehydration. Their persistence during the recovery periods is consistent with a sustained adjustment of secondary metabolism under abiotic stress [32,33,34,35,60]. At the genotype level, however, the magnitude of these responses varied substantially, confirming that broad treatment averages conceal contrasting patterns among genetic backgrounds.

Phenolic compounds and flavonoids can contribute to redox regulation, membrane protection, and stress signalling [32,33], and drought-associated changes in phenylpropanoid metabolism have been reported in several fruit crops [34,35,36,37,38,39,60]. In the present study, however, Folin–Ciocalteu and total-flavonoid assays quantify aggregate pools rather than individual compounds. These variables should therefore be interpreted as broad indicators of secondary-metabolite response and not as compound-resolved evidence for activation of specific phenylpropanoid or flavonoid pathways. Because individual phenolic and flavonoid metabolites can respond in different directions or with different amplitudes, similar aggregate pool sizes may conceal distinct compound-level stress signatures; targeted metabolomic studies in apple illustrate the additional resolution obtained when individual metabolites are resolved [16,61,62].

This analytical limitation does not eliminate the genotype-level information contained in the measurements. HL1282 and HL1579, for example, combined comparatively lower proline and MDA induction with stronger responses of total phenolics and total flavonoids, whereas genotypes such as ‘Idared’ showed a profile dominated more strongly by proline and MDA. Such contrasts help distinguish response strategies within the present marker panel, but targeted chromatographic or metabolomic profiling would be required to determine which individual phenolic and flavonoid compounds drive these differences.

### 3.5. Rehydration as an Active Component of the Stress Response

Rehydration was a distinct component of the experimental response rather than simply the absence of drought. During T3–T4 and T7–T8, most biochemical markers declined, but treatment means generally remained above the corresponding controls. This incomplete return was also evident in the genotype-specific temporal trajectories provided in the Supplementary Materials.

Recovery after drought involves restoration of redox homeostasis, turnover of osmolytes, repair of cellular structures, and readjustment of antioxidant metabolism [28,29,30,31,39,63,64,65]. At the molecular level, this recovery can involve rapid transcriptional reprogramming, chaperone-mediated restoration of protein homeostasis, proteolytic turnover of damaged proteins, repair of photosynthetic and membrane components, and re-establishment of metabolic and redox balance. Similar active post-drought recovery has been described in perennial species including olive, grapevine, and oak [30,31,39]. The persistence of elevated biochemical markers in the present study therefore supports the interpretation that rehydration represents an active metabolic phase and that the state reached after rewatering may influence the response to the next drought episode.

For genotype evaluation, recovery kinetics may be as informative as the peak stress response. A genotype showing moderate biochemical perturbation, limited MDA accumulation, and rapid return toward control values may represent a different strategy from one showing strong induction of multiple defence markers together with persistent oxidative damage. The present experiment was not designed to rank these strategies agronomically, but it demonstrates why peak values alone are insufficient for assigning drought tolerance.

The related physiological study [46] examined eight genotypes that are also included here—B11, ‘Gala (Galaval)’, HL308, HL827, HL2010, HL2350, ‘Idared’, and ‘Rubinstep’—and reported genotype-dependent differences in gas exchange, chlorophyll fluorescence, pigment content, water-use efficiency, and leaf water status. It also documented only partial physiological recovery after rehydration. These earlier observations provide useful context for the present biochemical results, but the two datasets differ in genotype coverage, replication, and measurement schedule; they were therefore not combined in a formal cross-domain statistical analysis.

### 3.6. Multivariate and Genotype-Specific Analyses Reveal Distinct Response Strategies

PCA, correlation analysis, and the genotype-specific heat maps demonstrate that the response to repeated drought cannot be reduced to a single biochemical variable. PC1, which explained 57.69% of the variance, represented a common gradient of overall biochemical response, whereas PC2 separated stronger contributions of total phenolics, POX, and CAT from profiles with greater contributions of proline, MDA, flavonoids, and SOD. This structure is consistent with the existence of different biochemical response combinations rather than a single linear axis of stress tolerance.

The separate T2 and T6 heat maps are particularly informative because averaging the two drought maxima alone would obscure cycle-dependent changes. Genotype-specific log_2_(Stress/Control) profiles showed that most genotypes intensified at least part of their biochemical response during the second drought, but the magnitude and composition of that change differed. HL827 showed one of the largest shifts from T2 to T6, driven especially by stronger MDA, CAT, and POX responses, whereas B11 showed stronger proline and SOD induction at T6 while MDA induction remained comparatively stable. In contrast, HL1651 maintained a broadly strong response across both drought maxima with relatively little overall amplification. Recurrent drought therefore modified not only response intensity but also the relative deployment of individual biochemical components.

The integrated clustering further resolved contrasting genotype-specific profiles. B11 combined comparatively low MDA induction with strong antioxidant-enzyme responses. HL1282 and HL1579 showed lower relative proline and MDA induction together with stronger total phenolic, total flavonoid, and selected enzyme responses. ‘Idared’, by contrast, showed high relative induction of proline and MDA, while several other genotypes occupied intermediate positions. These profiles do not identify tolerant and susceptible genotypes by themselves; rather, they provide a biochemical basis for selecting contrasting material for targeted physiological, metabolomic, and field validation.

The eight genotypes shared with the previous physiological study [46] permit a cautious qualitative comparison with previously reported physiological performance. The earlier study described B11 as relatively physiologically stable under water deficit; in the present dataset, B11 combined comparatively low MDA induction with strong antioxidant-enzyme responses. ‘Idared’, which was reported to show more pronounced physiological perturbation, exhibited strong proline and MDA induction here. The correspondence was not universal: HL2350 showed relatively stable physiological performance in the earlier study but an intermediate biochemical profile, while ‘Rubinstep’ showed marked physiological changes but only an intermediate-to-moderate biochemical profile across the present marker panel. These examples suggest that physiological and biochemical responses capture related but non-identical dimensions of stress performance. Because the physiological study included only eight of the fourteen genotypes and used a different measurement structure, no formal genotype-wide physiological–biochemical correlation was performed.

Accordingly, the present genotype-specific profiles should be interpreted as biochemical response strategies rather than direct measures of drought tolerance or susceptibility. Robust ranking for breeding purposes would require complete physiological data for the same genotype set together with recovery, growth, yield, and fruit-quality traits under both controlled and field conditions.

### 3.7. Implications for Genotype Evaluation and Apple Breeding

The practical value of the present study lies in showing that treatment-level averages conceal substantial genotype-specific variation. A complementary marker panel can distinguish whether a genotype responds predominantly through osmotic adjustment, antioxidant-enzyme activation, secondary-metabolite accumulation, or through combinations of these processes accompanied by oxidative damage. Such profiles are more useful for selecting contrasting genotypes for follow-up work than ranking material on the basis of proline or a single antioxidant enzyme alone.

Within the present dataset, potentially informative combinations include comparatively low MDA induction together with coordinated SOD, CAT, and POX responses, or lower proline/MDA induction accompanied by stronger total phenolic and total flavonoid responses. B11, HL1282, and HL1579 illustrate different versions of these profiles, whereas ‘Idared’ represents a contrasting pattern with strong proline and MDA induction. These patterns are best treated as hypotheses about response strategy rather than as evidence of superior or inferior drought tolerance.

The partial correspondence with previously reported physiological responses [46] reinforces this caution. The most informative next step will be to test the biochemical profiles against complete physiological, recovery, growth, yield, and fruit-quality datasets collected for the same genotypes. Such integration will determine whether the biochemical contrasts identified here have predictive value for agronomic performance under recurrent water limitation.

### 3.8. Limitations of the Study and Future Directions

This study was designed as a temporally dense, genotype-resolved comparison of seven biochemical markers rather than as comprehensive metabolomic or redox profiling. Total phenolics and total flavonoids were quantified as aggregate pools, and individual phenolic or flavonoid compounds were not identified. APX activity, ascorbate, glutathione, and other components of redox metabolism were also not measured. The results therefore support comparison of coordinated marker profiles but do not permit compound-specific or pathway-complete mechanistic conclusions. Recent multi-level studies in apple illustrate the additional mechanistic resolution that can be achieved by integrating biochemical measurements with physiological, molecular, anatomical, or metabolite-related information [61,62].

A second limitation is the controlled water regime, which enabled consistent comparison among fourteen genotypes but does not reproduce the combined environmental stresses encountered in orchards, including high temperature, variable vapour-pressure deficit, intense radiation, and heterogeneous soil water availability [48]. The polytunnel provided natural light and excluded direct precipitation. Microclimatic conditions were recorded during physiological assessments performed at the corresponding sampling dates in the same experimental facility (Table 1), but these point-in-time measurements do not represent continuous daily or phase-averaged records and therefore do not capture the full diurnal variability within each 14-day drought or rehydration period. In addition, the related physiological study [46] included only eight of the fourteen genotypes and used a different measurement structure. Physiological information was therefore used only as contextual support, and no genotype-wide physiological–biochemical correlation or tolerance index was constructed.

Future work should therefore proceed in complementary directions. First, targeted LC–MS/HPLC or broader metabolomic profiling should resolve the individual phenolic and flavonoid compounds underlying the aggregate responses detected here and should extend the redox panel to additional antioxidant components. Second, the biochemical response profiles should be tested against complete physiological, recovery, growth, yield, and fruit-quality datasets for the same genotypes, ideally under field conditions. Third, as reviewed by Ali et al. [66], emerging single-cell and spatial transcriptomic approaches can resolve cell type-specific stress programmes that are necessarily averaged in bulk-tissue biochemical measurements and could test whether the genotype-level heterogeneity identified here originates from contrasting responses of particular leaf cell types or tissues. Such integration will determine whether the contrasting biochemical strategies identified in the present experiment are predictive of stable performance under recurrent water limitation.

## 4. Materials and Methods

### 4.1. Plant Material and Experimental Setup

The experiment was conducted under partially controlled conditions in a foil-covered polytunnel at the Research and Breeding Institute of Pomology Holovousy Ltd., eastern Bohemia, Holovousy, Czech Republic (50.383629° N, 15.576902° E; 360 m a.s.l.). The facility provided natural light, excluded direct rainfall, and enabled controlled irrigation. The experimental system was established in early spring of 2023 using one-year-old nursery apple trees grafted onto M9 rootstock obtained from the Research and Breeding Institute of Pomology Holovousy Ltd., Holovousy, Czech Republic. Each tree was grown individually in a 30 L container filled with commercial RKS II substrate (AGRO CS Plc., Říkov, Czech Republic; pH 5.0–6.5; N 80–120 mg L^−1^, P 22–44 mg L^−1^, K 83–124 mg L^−1^), composed of 80% white peat and 20% black peat and supplemented with mineral soil (20 kg m^−3^). Containers were arranged at a spacing of 0.5 × 0.6 m. Additional physiological details of the same experimental system are reported in the related study [46].

Fourteen apple genotypes (*Malus domestica* Borkh.) were included: B11, ‘Gala (Galaval)’, HL53, HL308, HL827, HL1282, HL1311, HL1343, HL1579, HL1651, HL2010, HL2350, ‘Idared’, and ‘Rubinstep’. Five independent trees were used for each genotype × treatment combination, giving 140 experimental trees in total. Each tree occupied a separate container and constituted one biological replicate. Leaf samples collected from an individual tree were processed as that biological replicate; individual leaves and analytical aliquots were not treated as independent biological replicates.

Irrigation was supplied using a manually controlled drip system. Control plants received the full daily irrigation dose (15 L plant^−1^ day^−1^), whereas plants in the water-deficit treatment received 50% of this amount (7.5 L plant^−1^ day^−1^) during each drought period. The irrigation levels had been established in preliminary trials to provide adequate water to controls while inducing a reproducible, non-lethal water deficit in stressed plants. Substrate moisture was checked regularly to maintain the treatment contrast, although it was not continuously recorded by soil-moisture sensors. The imposed deficit produced visible wilting and, in the overlapping physiological experiment conducted in the same system, a clear decline in leaf water status [46]. During rehydration, stressed plants were returned to the full irrigation regime.

The experiment comprised two consecutive drought–rehydration cycles. Each drought period lasted 14 days and was followed by a 14-day rehydration period. Thus, the complete experimental sequence consisted of 14 days of drought, 14 days of rehydration, a second 14-day drought period, and a final 14-day rehydration period. During each rehydration period, stressed plants were returned to the full irrigation regime (15 L plant^−1^ day^−1^). The rehydration period was extended to 14 days compared with the shorter recovery period used in the preceding physiological experiment [46], because several physiological traits had shown only partial recovery after 7 days of rewatering. Biochemical analyses were performed at nine sampling dates: T0, common pre-treatment baseline; T1, beginning/early stage of the first drought period; T2, end of the first 14-day drought period; T3, beginning of the first rehydration period; T4, end of the first 14-day rehydration period; T5, beginning/early stage of the second drought period; T6, end of the second 14-day drought period; T7, beginning of the second rehydration period; T8, end of the second 14-day rehydration period.

Environmental conditions within the polytunnel were characterised using measurements recorded during physiological assessments performed at the corresponding sampling dates. Air temperature remained relatively stable at the time of assessment, ranging from 25.1 to 26.2 °C, relative humidity ranged from 50.0 to 57.0%, vapour-pressure deficit (VPD) from 1.43 to 1.62 kPa, and photosynthetic photon flux density (PPFD) from 710 to 760 µmol m^−2^ s^−1^ (Table 1). Values are reported as means ± SD. These measurements characterise conditions at the time of plant assessment and should not be interpreted as continuous daily means for the entire drought or rehydration periods.

### 4.2. Sample Collection and Storage

Ten fully developed leaves were collected from a comparable central position within the crown of each experimental unit at the same time of day (10:00–10:30 CET) to minimise circadian effects on the analysed biochemical parameters. Immediately after collection, the leaves were frozen in liquid nitrogen and stored at −80 °C until biochemical analysis, for a maximum of three months.

Each biological replicate was processed and analysed separately. For individual determinations, homogenised leaf material prepared from two leaves from each biological replicate was used.

### 4.3. Determination of Proline and Malondialdehyde Content

Free proline content was determined colorimetrically according to Bates et al. [67], with slight modifications. Approximately 0.5 g of frozen leaf tissue was homogenised in liquid nitrogen with 5 mL of 3% aqueous sulfosalicylic acid. The homogenate was centrifuged at 10,000× *g* for 10 min at 4 °C (Sigma 1-14K; Sigma Laborzentrifugen GmbH, Osterode am Harz, Germany). Two millilitres of the clear supernatant were mixed with 2 mL of ice-cold acetic acid and 2 mL of acidic ninhydrin reagent. The reaction mixture was incubated in a boiling water bath (100 °C) for 60 min and subsequently cooled. The chromophore was extracted with 5 mL of toluene, and after mixing, the upper organic phase was separated. Absorbance was measured at 520 nm using an Evolution 201 UV/VIS spectrophotometer (Thermo Scientific, Waltham, MA 02451, USA), with toluene as the blank. Proline concentration was determined from a calibration curve prepared using L-proline standards and expressed as µmol g^−1^ fresh weight (FW).

Membrane lipid peroxidation was assessed by determining malondialdehyde (MDA) content according to Kubes et al. (2024) [68]. Following reaction with thiobarbituric acid, the mixture was centrifuged at 11,000 rpm for 1 min at 4 °C (Sigma 1-14K; Sigma Laborzentrifugen GmbH, Germany). Absorbance was measured at 440, 532, and 600 nm using an Evolution 201 UV/VIS spectrophotometer (Thermo Scientific, Waltham, MA 02451, USA). MDA concentration was calculated using the equation described by Kubes et al. (2024) [68] and expressed as nmol g^−1^ FW.

### 4.4. Determination of Total Phenolics and Flavonoids

Total phenolic content was determined using the Folin–Ciocalteu method as described by Kubes et al. (2024) [68]. Absorbance was measured at 765 nm after 90 min using an Evolution 201 UV/VIS spectrophotometer (Thermo Scientific, Waltham, MA 02451, USA). Total phenolic content was calculated from a calibration curve prepared using gallic acid and expressed as mg gallic acid equivalents per gram of fresh weight (mg GAE g^−1^ FW).

Total flavonoid content was determined according to Kubes et al. (2024) [68]. Absorbance was measured at 415 nm against a blank using an Evolution 201 UV/VIS spectrophotometer (Thermo Scientific, Waltham, MA 02451, USA). Total flavonoid content was calculated from a calibration curve prepared using quercetin and expressed as mg quercetin equivalents per gram of fresh weight (mg QE g^−1^ FW).

### 4.5. Determination of Antioxidant Enzyme Activities

Enzyme extracts were prepared according to Kubes et al. (2024) [68], and enzyme activities were normalised to soluble protein content. Soluble protein content was determined using the same methodological procedure. Extracts were centrifuged at 2350× *g* for 25 min at 4 °C, and the resulting supernatant was used for enzyme activity assays.

Superoxide dismutase (SOD; EC 1.15.1.1) activity was determined spectrophotometrically based on inhibition of the photochemical reduction of nitroblue tetrazolium (NBT) at 560 nm. Catalase (CAT; EC 1.11.1.6) activity was determined by monitoring the decrease in H_2_O_2_ absorbance at 240 nm resulting from its enzymatic decomposition. Peroxidase (POX; EC 1.11.1.7) activity was determined spectrophotometrically by monitoring guaiacol oxidation at 470 nm. All enzyme assays were performed according to Kubes et al. (2024) [68].

### 4.6. Statistical Analysis

Statistical analyses were performed using Statistica 12 (StatSoft Inc., Tulsa, OK, USA) at the level of individual biological replicates (one tree = one biological replicate; n = 5 per genotype × treatment combination at each sampling date). Means and standard deviations were calculated for all measured parameters. The effects of genotype, experimental treatment, sampling date, and their two- and three-factor interactions were assessed using three-way analysis of variance (ANOVA). Effect sizes were expressed as partial η^2^ (partial eta squared). Differences between the control and stress treatments at individual sampling dates were assessed using Tukey’s HSD post hoc test. Differences were considered statistically significant at *p* < 0.05.

Relationships among the analysed biochemical markers were assessed using Pearson’s correlation analysis across the individual biological observations. PCA was likewise performed on the 1260 individual biological observations (14 genotypes × 2 treatments × 9 sampling dates × 5 independent trees), using the seven biochemical variables after Z-score standardisation. The treatment × sampling-time points shown in Figure 6 are centroids calculated only for graphical presentation; they were not the observation units used to fit the PCA. For genotype-specific drought-maxima analyses, mean values were first calculated for each genotype × treatment at T2 and T6, log_2_(Stress/Control) response ratios were calculated for each marker, and the T2 and T6 response ratios were averaged. These integrated genotype profiles were then standardised as Z-scores and subjected to hierarchical clustering using Euclidean distance and Ward’s method, with a four-cluster solution used for interpretation.

Cluster robustness was assessed by bootstrap consensus resampling (1000 iterations). Within each genotype × treatment × drought-maximum combination, the five biological replicates were resampled with replacement; log_2_ response ratios, integrated T2–T6 profiles, Z-score standardisation, and Ward clustering were recalculated at each iteration. Stability was summarised as the mean pairwise probability that members of each original cluster were assigned to the same cluster across bootstrap iterations. The number of clusters was evaluated for solutions comprising two to six clusters using the average silhouette coefficient. The four-cluster solution provided the best compromise between cluster separation, stability, and biological interpretability and was therefore retained for subsequent interpretation. Correlation matrices, PCA plots, heat maps, and hierarchical clustering dendrograms were generated in Python (version 3.13) (Python Software Foundation, Wilmington, DE, USA) using the Seaborn (version 0.13) and Matplotlib (version 3.11) libraries. Bootstrap procedures used NumPy (version 2.3), SciPy (version 1.16), and scikit-learn (version 1.8).

## 5. Conclusions

Repeated water deficit induced coordinated but distinctly genotype-dependent biochemical responses in apple plants. Although the treatment-level means showed a common temporal pattern, genotype-specific analysis at the two drought maxima revealed substantial differences in the relative contributions of osmotic adjustment, oxidative damage, enzymatic antioxidant defence, and secondary metabolism. The strongest overall response occurred at the end of the second drought cycle (T6), but the extent to which individual markers intensified from T2 to T6 differed among genotypes, indicating that recurrent water deficit altered the balance of biochemical response components in a genotype-specific manner.

Proline showed the largest mean relative increase under water deficit, but its positive correlation with MDA demonstrates that high proline accumulation should not be interpreted independently as evidence of greater drought tolerance. Genotype-specific profiles further showed that some genotypes, such as B11, combined comparatively low MDA induction with strong antioxidant responses, whereas HL1282 and HL1579 showed lower proline and MDA induction together with stronger phenolic- and flavonoid-related responses. Other genotypes, including ‘Idared’, showed stronger proline and MDA induction relative to several antioxidant-related traits.

Rehydration reduced most biochemical markers, but recovery to control levels was generally incomplete. The ability to recover after water deficit therefore remains an important component of the overall response to recurrent drought. For the eight genotypes shared with the previous physiological study, qualitative comparison indicated partial correspondence between biochemical profiles and physiological performance, while also showing that the two response levels are not interchangeable.

Overall, the results support the use of coordinated biochemical profiles, rather than individual biomarkers, for describing genotype-specific responses to recurrent water limitation. The present marker panel provides a basis for selecting contrasting genotypes for more detailed physiological, metabolomic, growth, yield, and fruit-quality evaluation. Targeted analysis of individual phenolic and flavonoid compounds will be particularly important for increasing the mechanistic resolution of future studies.

## Figures and Tables

**Table 1:** Environmental conditions recorded during plant assessments throughout two consecutive drought–rehydration cycles. Values are means ± SD. RH, relative humidity; VPD, vapour-pressure deficit; PPFD, photosynthetic photon flux density.

PPFD (µmol m^−2^ s^−1^)	VPD (kPa)	RH (%)	Air Temperature (°C)	Phase	Sampling
720 ± 45	1.43 ± 0.03	55.0 ± 4.3	25.1 ± 0.3	Baseline	T0
730 ± 50	1.52 ± 0.05	54.0 ± 5.2	25.7 ± 0.5	Drought I	T1
710 ± 48	1.57 ± 0.04	55.0 ± 4.4	25.5 ± 0.5	Drought I maximum	T2
740 ± 43	1.43 ± 0.03	56.0 ± 3.6	25.4 ± 0.4	Rehydration I	T3
750 ± 47	1.51 ± 0.05	57.0 ± 5.1	25.6 ± 0.3	End of rehydration I	T4
760 ± 50	1.52 ± 0.03	54.0 ± 5.2	25.5 ± 0.3	Drought II	T5
730 ± 51	1.59 ± 0.04	55.0 ± 4.8	25.7 ± 0.5	Drought II maximum	T6
750 ± 48	1.60 ± 0.05	52.0 ± 3.9	26.0 ± 0.4	Rehydration II	T7
760 ± 46	1.62 ± 0.04	50.0 ± 4.7	26.2 ± 0.4	Recovery	T8

## Data Availability

The data supporting the findings of this study are contained within the article and its Supplementary Materials. The minimal dataset supporting the results of this study is provided as Supplementary Dataset S1.

## Supplementary Materials

The following supporting information can be downloaded at: https://www.mdpi.com/article/10.3390/plants15172701/s1. Figure S1. Genotype-specific temporal dynamics of proline content in control and drought-stressed apple plants during two consecutive drought–rehydration cycles. Values represent means ± SD of five biological replicates for each genotype. T0 represents the common pre-treatment baseline and was therefore not included in genotype-specific post hoc comparisons between control and stress treatments. Asterisks indicate significant differences between treatments at T1–T8 based on Tukey’s HSD test (*p* < 0.05, *p* < 0.01, *p* < 0.001). Figure S2. Genotype-specific temporal dynamics of malondialdehyde (MDA) content in control and drought-stressed apple plants during two consecutive drought–rehydration cycles. Values represent means ± SD of five biological replicates for each genotype. T0 represents the common pre-treatment baseline and was therefore not included in genotype-specific post hoc comparisons between control and stress treatments. Asterisks indicate significant differences between treatments at T1–T8 based on Tukey’s HSD test (*p* < 0.05, *p* < 0.01, *p* < 0.001). Figure S3. Genotype-specific temporal dynamics of superoxide dismutase (SOD) activity in control and drought-stressed apple plants during two consecutive drought–rehydration cycles. Values represent means ± SD of five biological replicates for each genotype. T0 represents the common pre-treatment baseline and was therefore not included in genotype-specific post hoc comparisons between control and stress treatments. Asterisks indicate significant differences between treatments at T1–T8 based on Tukey’s HSD test (*p* < 0.05, *p* < 0.01, *p* < 0.001). Figure S4. Genotype-specific temporal dynamics of catalase (CAT) activity in control and drought-stressed apple plants during two consecutive drought–rehydration cycles. Values represent means ± SD of five biological replicates for each genotype. T0 represents the common pre-treatment baseline and was therefore not included in genotype-specific post hoc comparisons between control and stress treatments. Asterisks indicate significant differences between treatments at T1–T8 based on Tukey’s HSD test (*p* < 0.05, *p* < 0.01, *p* < 0.001). Figure S5. Genotype-specific temporal dynamics of peroxidase (POX) activity in control and drought-stressed apple plants during two consecutive drought–rehydration cycles. Values represent means ± SD of five biological replicates for each genotype. T0 represents the common pre-treatment baseline and was therefore not included in genotype-specific post hoc comparisons between control and stress treatments. Asterisks indicate significant differences between treatments at T1–T8 based on Tukey’s HSD test (*p* < 0.05, *p* < 0.01, *p* < 0.001). Figure S6. Genotype-specific temporal dynamics of total phenolic content in control and drought-stressed apple plants during two consecutive drought–rehydration cycles. Values represent means ± SD of five biological replicates for each genotype. T0 represents the common pre-treatment baseline and was therefore not included in genotype-specific post hoc comparisons between control and stress treatments. Asterisks indicate significant differences between treatments at T1–T8 based on Tukey’s HSD test (*p* < 0.05, *p* < 0.01, *p* < 0.001). Figure S7. Genotype-specific temporal dynamics of total flavonoid content in control and drought-stressed apple plants during two consecutive drought–rehydration cycles. Values represent means ± SD of five biological replicates for each genotype. T0 represents the common pre-treatment baseline and was therefore not included in genotype-specific post hoc comparisons between control and stress treatments. Asterisks indicate significant differences between treatments at T1–T8 based on Tukey’s HSD test (*p* < 0.05, *p* < 0.01, *p* < 0.001). Table S1. Results of three-way ANOVA for the seven biochemical parameters, including the effects of genotype, treatment, sampling date, and their interactions, with *p*-values and partial η^2^. Table S2. Mean biochemical parameters for individual genotypes, treatments, and sampling dates (mean ± SD; n = 5 biological replicates). Table S3. PCA loadings and variance explained by the principal components. Table S4. Genotype-specific standardised biochemical responses used for hierarchical clustering, including cluster membership. Table S5. Bootstrap consensus stability of the four hierarchical clusters, including mean and minimum within-cluster pairwise co-clustering probabilities based on 1000 resamples.
