# Supplementary material for: Genotype-Specific Biochemical Responses of Apple Trees to Repeated Drought–Rehydration Cycles
Source: Plants (Basel). 2026 Sep 2;15(17):2701. doi: 10.3390/plants15172701 (PMC13567614; doi:10.3390/plants15172701)
Supplement: Supplementary file 1 [file plants-15-02701-s001.zip › Figure_S6_Total_phenolics.pdf]

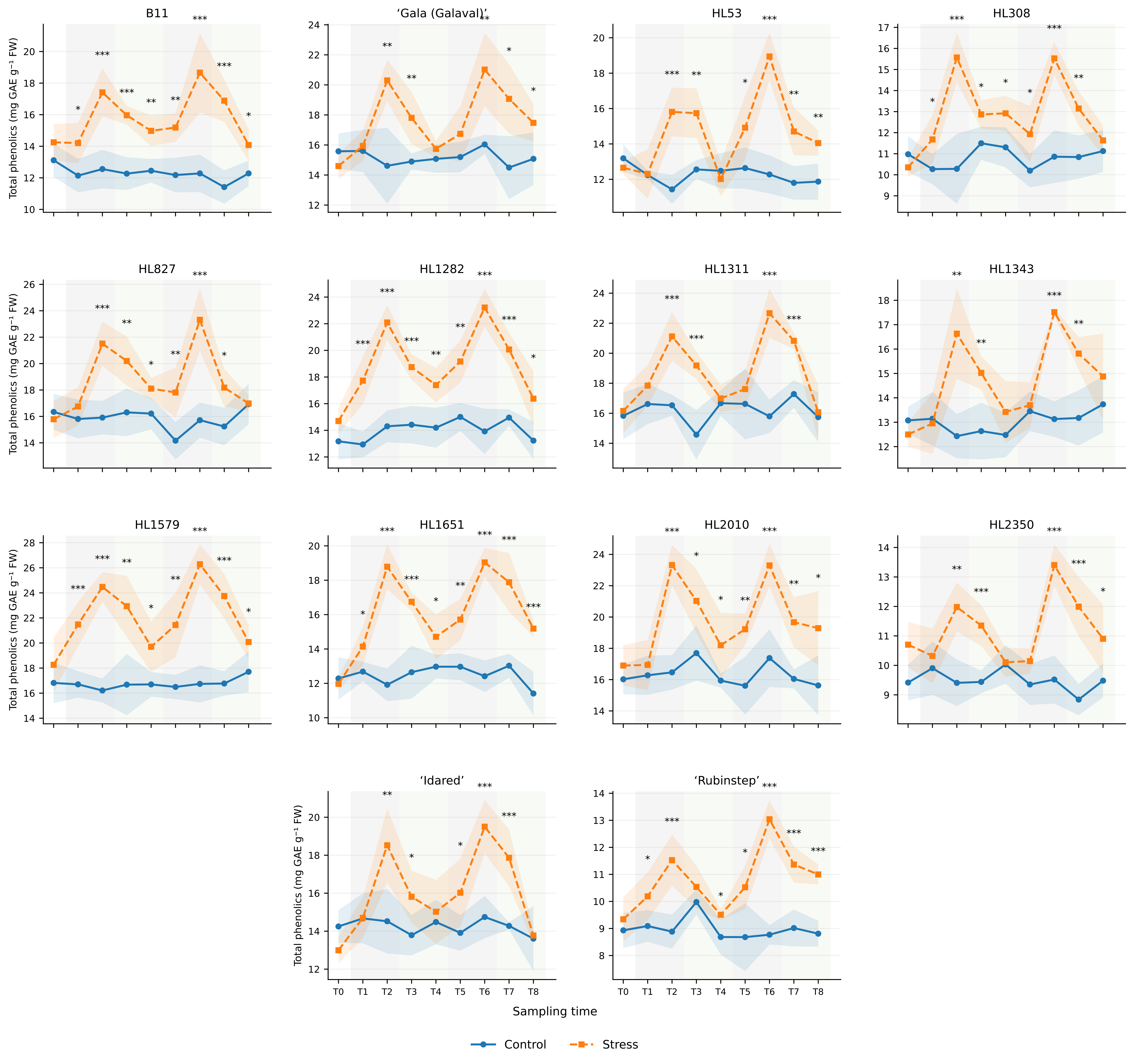

B11
'Gala (Galaval)'
HL53
HL308
HL827
HL1282
HL1311
HL1343
HL1579
HL1651
HL2010
HL2350
'Idared'
'Rubinstep'
Total phenolics (mg GAE g⁻¹ FW)
T0
T1
T2
T3
T4
T5
T6
T7
T8
Sampling time
Control
Stress
